# Enhancing synthetic vinasse treatment efficiency using an integrated UASB-Modified Bardenpho Process

**DOI:** 10.1186/s40643-024-00830-z

**Published:** 2024-12-11

**Authors:** Afsaneh Mazaheri, Mohamad Reza Doosti, Mohammad javad Zoqi

**Affiliations:** Department of Civil and Environmental Engineering, Faculty of Engineering, University of Birjand, P.O.Box: 97175/615, Birjand, Iran

**Keywords:** Wastewater treatment, Vinasse, Anaerobic treatment, UASB reactor, Modified Bardenpho processes

## Abstract

Vinasse poses considerable environmental problems due to its complex composition of organic matter, minerals, and toxic compounds. If discharged into the environment without treatment, it can cause adverse impacts on ecosystems. This research investigated the effectiveness of an integrated treatment system involving an upflow anaerobic sludge blanket (UASB) reactor and the modified Bardenpho process (MBP) for purifying synthetic vinasse. The study lasted for 167 days, during which the integrated UASB-MBP system processed untreated synthetic vinasse with organic loading rates (OLR) ranging from 1.6 to 12.5 kgCOD/m^3^ day. The UASB-MBP system impressively achieved a COD removal efficiency of 99.41%. Removal efficiencies of approximately 98.14, 99.91, and 99.63% were also achieved for total nitrogen (TN), total phosphorus (TP) and total ammonium (NH_4_^+^-N), respectively. The final discharge was 51.06 mg/L. The concentrations of NH_4_^+^-N and TN in the outflow of the settlement tank were 0.8–1.2 mg/L and 5.1–7.9 mg/L, respectively. Optimal performance was achieved when the HRT and nitrate recycle ratio were 15.5 h and 200%, respectively. The temperature was kept in the mesophilic range (33–35 °C) during the experiments. These results underscores the potential of the integrated UASB reactor and modified Bardenpho process to provide an effective and eco-friendly approach for concurrent removal of COD and nutrients from vinasse treatment, offering broad prospects for implementation in wastewater treatment.

## Introduction

Vinasse is considered one of the 17 most polluting wastewater, with a pollution potential 100 times greater than domestic sewage (Montaño Saavedra et al. 2024; Türk and Arslanoğlu 2024). Vinasse is characterized by high-strength properties, including substantial phenolic compounds, which have dark brownish color with acidic pH in range (3.5–5.0), a high Biochemical Oxygen Demand (BOD) ranging from 15,000–65,000 mgO_2_/L, a high Chemical Oxygen Demand (COD) ranging from 70,000–150,000 mg/L, a total solids (TS) concentration range of 10,780–38,680 mg/L, and a carbohydrates concentration range (4.6–10.2 g/L) (Cruz-Salomón et al. 2017; Utami et al. 2020; de Santi Caraça et al. 2023; Tibúrcio Neto et al. 2024). Each liter of ethanol production generates approximately 10 to 18 L of vinasse (de Brito et al. 2024; Madaleno et al. 2024; Montaño Saavedra et al. 2024). Globally, approximately 22.4 gigaliters (GL) of vinasse is produced annually, with potential biogas generation of 407.68 GL (Parsaee et al. 2019). Vinasse can contaminate water and harm aquatic ecosystems if it is discharged to the environment without treatment (Hurtado et al. 2021; de Brito et al. 2024). Therefore, it is essential to treat vinasse for reducing pollution while maintaining its fertilization and biogas production benefits (Azevedo-Santos et al. 2024).

Various chemical, physical, and biological techniques have been implemented to treat vinasse to meet regulatory discharge requirements, which specify COD ≤ 10 mg/L and NH₄⁺-N ≤ 25 mg/L in the vinasse effluent (Ong 2018). Biological methods, especially anaerobic treatment, are cost-effective and environmentally friendly, excelling in reducing organic loads and biogas production (Yang et al. 2021; Cisneros de la Cueva et al. 2024; Li et al. 2024). Numerous anaerobic reactors have been used for vinasse treatment, such as Upflow anaerobic sludge blanket (UASB) (Barbosa et al. 2022; Alves et al. 2023). UASB reactors are efficient in COD removal, biogas production (> 50%), and considerable microbial biomass removal (30,000–80,000 mg/L), but don’t fully remove organic matter and nutrients such as ammonia and phosphate from high-strength wastewater (Yanqoritha et al. 2018; Gunes et al. 2019; Magdalena et al. 2020; Mikucka and Zielińska 2020). Thus, a post-treatment step after UASB reactor may be needed to remove residual nutrients and organic substances to produce a high-quality effluent.

Biological nutrient removal (BNR) techniques are often used for the post-treatment of UASB reactor effluent to eliminate nutrients. Common BNR processes include the Modified Ludzack-Ettinger (MLE), anaerobic-anoxic–oxic (A_2_/O), anoxic–oxic (A/O), and Modified Bardenpho (MBP) (Banayan Esfahani et al. 2019; Jafarinejad 2021; Abyar et al. 2022). Wang et al. compare the performance of the AO, A_2_O, and Modified Bardenpho process (MBP). Their results show that while the AO process primarily targets phosphorus removal, MBP can remove both TN and TP simultaneously and has a superior COD removal efficiency (Wang et al. 2023). This has also been proven by other study (Tian et al. 2022). Therefore, combining a UASB reactor with the Modified Bardenpho process provides economic benefits and improves COD removal by extending nitrification sludge age and utilizing vinasse carbon, making it a preferred choice for vinasse treatment.

However, no investigation have been conducted on treating vinasse using an integrated UASB-MBP system. The most frequently integrated UASB-biological systems used for vinasse treatment involve the combination of UASB with various bioreactors, such as AO, membrane bioreactors (MBR), reverse osmosis (RO), activated sludge (AS), and anaerobic packed bed reactors (APBR) (Ma et al. 2013; Cabrera-Díaz et al. 2017; Ashrafi et al. 2019; Reis et al. 2019; Procópio et al. 2022; Alves et al. 2023; de Santi Caraça et al. 2023; De Carvalho Filho et al. 2024; Mazaheri et al. 2024; Tibúrcio Neto et al. 2024). Therefore, this research evaluated the efficiency of an integrated UASB-MBP system for treating synthetic vinasse at the laboratory scale. Synthetic samples allow precise control over variables and provide an accurate study of the wastewater treatment process. The results obtained from these laboratory experiments will form the basis for conducting large-scale experiments at the industrial level.

The aim of this research were: (1) to evaluate the Modified Bardenpho process as a secondary treatment method to enhance COD removal, (2) To examine the integrated system's ability to remove NH_4_^+^-N, TN, TP in synthetic vinasse treatment, and (3) To investigate all parameters affecting system performance, including optimal organic loading rate (OLR), pH variation, HRT, and Vup in each step of synthetic vinasse purification.

## Materials and methods

### Reactor organization

#### Configuration of integrated UASB–MBP

**Fig. 1:**
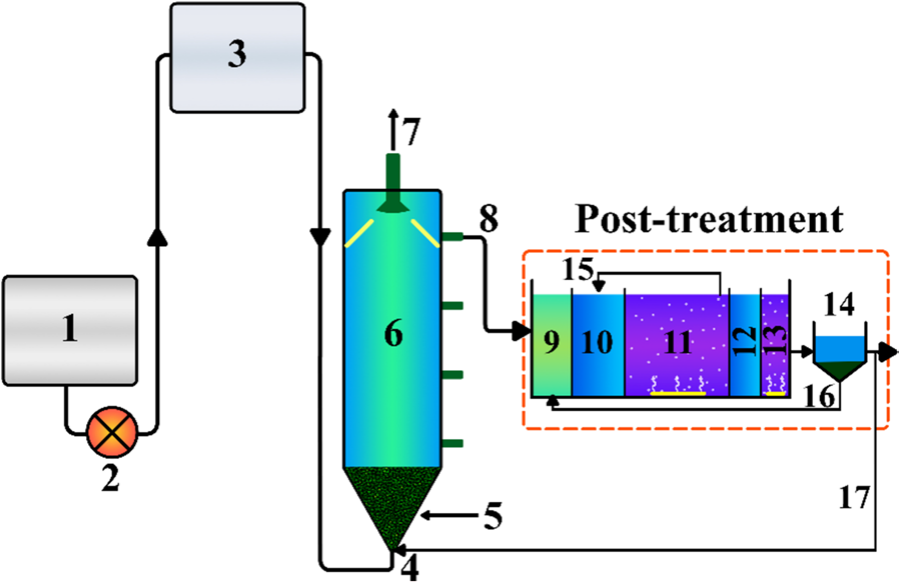
A schematic of the system. (1) Feed tank, (2) Pump, (3) Regulation tank, (4) Influent, (5) Sludge blanket, (6) UASB reactor, (7) Biogas output of UASB reactor, (8) UASB-Effluent, (9) Anaerobic reactor, (10) First anoxic reactor, (11) First aerobic reactor, (12) Second anoxic reactor, (13) Second aerobic reactor, (14) Setteling tank (15) Nitrification reflux, (16) sludge reflux, and (17) Effluent recirculation

Figure 1 present a schematic illustration of the integrated UASB-MBP system. The system comprised a 43 L UASB reactor (20 × 20 × 120 cm) and a modified Bardenpho processes (MBP) with a total volume of 24 L. A 5.7 L cylindrical settlement tank (with an internal diameter of 11 cm and a height of 60 cm) was used to settle the biological sludge, allowing the production of a clear effluent. A 20 L feed tank and a 20 L regulating tank were utilized to adjust pH and maintain a continuous inlet flow rate.

The UASB reactor operated with a working volume of 33 L. Four sampling faucets were installed along the UASB reactor wall, spaced 30 cm apart. Additionally, two 45° baffles were mounted on the upper part of the reactor to differentiate between the gas and liquid phases.

The modified Bardenpho bioreactor consists of five reaction tanks and a settling tank. The five reactors comprised a anaerobic tank, a primary anoxic tank, a primary aerobic tank, a secondary anoxic tank, and a secondary aerobic tank. The denitrification zone size should occupy 30% of the whole volumetric capacity of the reactor (Maciołek et al. 2021). Hence, the volume of the primary anaerobic reactor was 4 L, and the volume of the primary anoxic tank was 7 L. The volumes of the remaining three reactors were 10, 2, and 2 L, respectively.

Figure 1 shows the wastewater is pumped into the conic part of the UASB reactor at a rate of about 100 L/min using a pump (Model: Zagros, Tabriz Electricity, Iran). Then, the UASB-purified outflow fed into the MBP bioreactor. To ensure that sufficient aeration was maintained during the aerobic phase inside the aerobic reactors, an air pump (Model: RS513, RS Electrical, China) delivering 2.2 L/min of air volume was used. The final stage involved the settling tank, where the effluent was discharged. The sludge at the bottom of the settling tank refluxes to the primary anaerobic reactor at an air flow rate of 11 L/min (Model: MD-10R, magnetic pump, China). A pump (Model: A YZII15, peristaltic pump, China) was used to transfer nitrification products from the primary aerobic reactor to the primary anoxic reactor.

#### Characteristics of wastewater

The nature of the feedstock, used in the ethanol production process, influences the properties of vinasse (España-Gamboa et al. 2011). The mean elemental concentration of synthetic vinasse was determined using data from synthetic wastewater studies conducted by Nogueira et al. (Nogueira et al. 2021). The detailed characteristics of the synthetic vinasse are illustrated in Table 1.

**Table 1:** Characterization of the synthetic vinasse

Integrant	Concentration
Total COD (mg/L)	6000
Sucrose (mg/L)	3452
Ethanol (mg/L)	1040
Acetic acid (mg/L)	248
Propionate (mg/L)	135
Butyrate (mg/L)	152
Phenol (mg/L)	1.5
Sulfate (mg ${\text{SO}}_{4}^{2-}$/L)	1923
Nitrogen (mg TKN/L)	205
Total phosphorus (mg ${\text{PO}}_{4}^{3+}$/L)	124
Potassium (mg ${\text{K}}^{3+}$/L)	519
Calcium (mg ${\text{Ca}}^{2+}$/L)	223.5
Sodium (mg ${\text{Na}}^{+}$/L)	102.5
Magnesium (mg ${\text{Mg}}^{2+}$/L)	84
Iron (mg ${\text{Fe}}^{2+}$/L)	10.9
Manganese (mg ${\text{Mn}}^{2+}$/L)	1
Zinc (mg ${\text{Zn}}^{2+}$/L)	* < 0.1
Copper (mg ${\text{Cu}}^{2+}$/L)	* < 0.1

*These amounts are less than the 0.1

Various components, such as butyrate, macronutrients, and micronutrients were incorporated to produce synthetic vinasse resembling natural vinasse. Butyrate and propionate are crucial components for the anaerobic degradation process. The incorporation of macronutrients, including nitrogen, phosphorus into the UASB reactor enhances adsorption, adhesion, and the proliferation of sludge granulation. The inclusion of micronutrients, such as calcium, and magnesium in wastewater has been shown to enhance methanogenic activity, increase the granulation rate, and improve the sedimentation capacity of sludge (Boonsawang et al. 2008; Ali et al. 2021). Furthermore, nitrogen positively impacts the restoration of microorganisms' cell structure. Hence, if nitrogen levels are notably low, microbial growth may be inhibited, potentially disrupting the methanation process (Sydney et al. 2014). Synthetic vinasse had a pH of approximately 4.0 ± 0.3.

#### Inoculation

Before initializing the operation of the integrated UASB-MBP system, all reactors underwent inoculation. The granular sludge used in this investigation was procured from the wastewater treatment division of a slaughterhouse facility located in Birjand, Iran. Inoculation of integrated UASB-MBP system with high-quality sludge accelerates the development of granules, resulting in enhanced stability during the start-up stage and an increased ability to adjust to higher OLR. The features of the sludge is described in Table 2.

**Table 2:** Characteristics of the sludge

Component	Unit	Concentration
MLVSS	mg/L	1325 ± 2.3
MLSS	mg/L	1736 ± 2.1
MLVSS/MLSS		0.76 ± 0.03
VSS	mg/L	123 ± 2.5
TSS	mg/L	195 ± 2.2
VSS/TSS		0.63 ± 0.02
SVI	mL/g	112 ± 1.2

Important indicators such as SVI, MLSS/MLVSS, and VSS/TSS are used to assess the quality of sludge. SVI of a high-quality sludge typically falls within the range of 100–150 mL/g. In this research, the SVI of the sludge was approximately 112 (Table 2). Also, the MLVSS/MLSS ratio ought to exceed 0.75 (Fan et al. 2015), and optimal VSS/TSS ratio should approximately be one (Mazaheri et al. 2024). Thus, the sludge utilized for seeding is deemed to be of good quality.

To ensure proper inoculation of the UASB reactor, it is recommended to fill 10–30% of its volume with granular sludge (Mainardis et al. 2020). Hence, anaerobic sludge filled up 10% of the UASB reactor’s capacity. Also, 20% of the modified Bardenph reactor’s capacity was allocated to anaerobic granular sludge and aerobic granular sludge. Because of low carbon to nitrogen ratio of the vinasse, supplementary substances such as animal manure, potash fertilizers, nutrients need to be added to increase the biogas production (Parsaee et al. 2019). Therefore, synthetic wastewater comprising a sucrose solution, urea, and potash fertilizer as carbon and nitrogen sources was introduced into the integrated UASB-MBP system to improve bioactivity and granular sludge formation. The sludge and chemical fertilizer were mixed in a proportion of 5 parts sludge to 1 part chemical fertilizer. The UASB reactor received synthetic vinasse after granular sludge had been developed and bacteria had acclimated to the noxious conditions. The UASB reactor outflow was then directed to the post-treatment modified Bardenpho bioreactor for further purification of the outflow.

#### Laboratory operations

The combined UASB-MBP system lasted for 167 days, with laboratory procedures performed in two stages, UASB reactor operation and MBP operation. Each stage was divided into three periods: start-up, adaptation, and stability (Barcelos et al. 2022; Mazaheri et al. 2024). UASB reactors alone are insufficient for removing residual organic matter, nutrients, and pathogens. Therefore, additional post-treatment is typically required after anaerobic processing to ensure the effluent complies with discharge standard (Daud et al. 2018). Given the high toxicity of compounds in vinasse, it is essential to reduce COD concentration to around 1000 mg/L and to degrade hard-to-biodegrade compounds using anaerobic microorganisms. This conversion into simpler, less complex compounds enhances the effectiveness of aerobic treatment in MBP reactors as a post-treatment step. Inoculation of the MBP was initiated later than UASB reactor. When the effluent of the UASB reactor became stable during its stability phase, it was then fed into the MBP. In addition, it helps to prevent an increase in sludge age of the MBP's aerobic reactors. The operations parameters of the Integrated UASB-MBP system in each stage were evaluated.

• Stage 1 (UASB reactor operation)

The UASB reactor operated for 136 days, with the operation divided into three periods: start-up, adaptation, and stability. In the start-up stage, the sludge blanket was inoculated and formed at the lower section of the UASB reactor for a period of 30 days. Then, the sludge blanket underwent a gradual exposure to synthetic vinasse, allowing microorganisms to acclimate to the new conditions. Failure to gradually expose the sludge to wastewater may result in organic loading shocks, which have the potential to induce sludge degradation. Hence, OLR was progressively raised from 0.5 to 1.5 kgCOD/m^3^.day over a period of 5 days. During the adaptation phase, synthetic vinasse was continuously fed to the UASB reactor, with the OLR ranging from 1.52 to 6.2 kgCOD/m3·day over 47 days, until the reactor reached a stable state. The highest COD removal was identified in the stability stage, as the OLR was incrementally raised from 6.2 to 12.5 kgCOD/m^3^.day over the course of the 54 day period. Anaerobic microorganisms experience inhibition growth when the NH_4_^+^-N concentration exceeds 150 mg/L (Liu et al. 2015). Therefore, a portion of the bio-treatment wastewater was circulated back to the reactor so that the vinasse becomes diluted, enhancing the COD removal efficiency of the UASB process. This approach reduced the impeding effect of the elevated NH_4_^+^-N levels on anaerobic bacteria. Furthermore, optimizing the operational conditions of the modified Bardenpho process led to improved removal efficiencies of total ammonium and nitrogen. Simultaneously with the increase in loading rate, the upflow velocity (*V*_up_) was gradually raised from 0.43 to 0.75 m/h. Throughout the three phases of the UASB reactor, the pH fluctuated from 6.6 to 7.6.

• Stage 2 (MBP operation)

The modified MBP operation took place over a period of 32 days, while the inoculation and granular sludge formation required 10 days. The modified Bardenpho bioreactor was then acclimatized to UASB-treated wastewater. The acclimation process began with synthetic vinasse COD concentration of 500 mg/L and was incrementally increased to about 1600 mg/L during a 10 days. Once the microorganisms had acclimated to the new environment, the MBP bioreactor received a continuous supply of UASB-effluent, while the OLR was steadily raised from 0.5 to 3.8 kgCOD/m^3^.day during a 12 days. The influent (UASB-effluent) flow rate was 1000 m^3^/h, with a return activated sludge (RAS) ratio of 100%, and a nitrate recycle ratio of 200%. The pH was in the range of 6.5 to 7.9. The dissolved oxygen (DO) was controlled and kept between 4–5.5 mg/L and less than 0.5 mg/L in aerobic and anoxic reactors, respectively.

### Sampling

Triplicate samples of effluent and biogas were collected to assess the efficiency and stability of each reactor. Samples of the UASB reactor were collected from the uppermost sampling faucet during five-day cycles. Sludge specimens were gathering from the exit point of the conic portion, whereas all biogas samples were collected from the gas departure point. The MBP bioreactor specimens were collected from each of the five reactors during 3 day cycles.

### Analytical methods

#### COD removal efficiency

Prior to dilution with refining water (10% v/v), the samples were filtered using filter paper. Subsequently, vials containing half of the diluted samples (1.5 ml) and the catalyst (3.5 ml) were filled with 2 ml of the diluted samples and heated at 150 °C for two hours using a Thermoblock (Model: BIO TDB-100, BIOSAN, Lettland). The samples were analysed utilizing a spectrophotometer (Model: sq2800, UNICO, USA). Equation 1 was utilized to determine the COD removal efficiency (De Carvalho Filho et al. 2024):

1 $${\text{COD removal (\% )}} = \frac{{COD_{i} - COD_{e} }}{{COD_{i} }} \times 100$$

where COD_*i*_ represents the inflow COD concentration, and COD_*e*_ indicates the outflow COD concentration.

#### OLR

The OLR indicates the amount of organic matter in the bioreactor wastewater that is either soluble or suspended. The OLR is calculated using the HRT and the COD concentration. Equation 2 is used to compute the OLR (Mariraj Mohan and Swathi 2022):

2 $${\text{OLR}} = \frac{{QS_{0} }}{V}$$

where OLR is the organic loading rate (kgCOD/m^3^ day), Q is the flow rate (m^3^/d), S_0_ is the input flow concentration (mg/L), and V is the reactor volume (m^3^).

#### Nitrate removal efficiency

A spectrophotometer (Model: sq2800, UNICO, USA) with the wavelength 430 nm was utilized to measure nitrate. The nitrogen removal efficiency (g) in the modified Bardenpho system was determined by Eq. 3 (Hassan et al. 2020):

3 $${\text{The removal efficiency of Nitrates reduction (\% )}}\, = \,\left( {\frac{{Nitrate_{in} - Nitrate_{out} }}{{Nitrate_{in} }}} \right)\, \times \,100\%$$

where “Nitrate _in_” is the initial nitrate concentration, and “Nitrate _out_” denotes the concentration of nitrate at the time, t.

#### HRT

HRT indicates the time in hours required for wastewater to flow across the reactor. The HRT was determined by Eq. 4 (Mazaheri et al. 2024):

4 $${\text{HRT (h)}} = \frac{V}{Q}$$

where V denotes the tank's volume, and Q represents the flow rate.

#### pH

The pH was assessed using a digital pH meter (model 86502, AZ Instrument Corp., Taiwan) to measure acidity.

The Integrated UASB-MBP system was kept at a steady temperature in the mesophilic range (33–35 °C) throughout all phases with the help of an electric heater (Model: 50-Watt, RS, China). Ammonium (NH_4_^+^-N), total nitrogen (TN), and total phosphorus (TP) were measured using standard methods (Baird et al. 2017). DO was assessed using a DO portable device (Model: AD630, EDVA, Romania). All experiments were carried out using the standard methods described by the APHA (Baird et al. 2017).

## Results and discussion

### Assessment of the UASB performance

The UASB reactor demonstrated high COD removal efficiency at its maximum loading capacity over the course of 136 days of operation. The capability of the UASB reactor to remove COD and its maximum loading capacity were determined throughout the 136-day period. The performance of the UASB reactor is divided into three periods: start-up, adaptation, and stability. The UASB reactor perfprmance is summarised in Table 3.

**Table 3:** Summary of the UASB reactor's performance

Stage	Days	Influent	OLR (KgCOD/m^3^.day)	COD Removal (%)	HRT (h)	upflow velocity (*V*up) (m/h)
Start-up phase	0–30	Synthetic wastewater	0.5–4	98	24	0.43–0.57
Adaptation phase	30–82	Synthetic vinasse	1.5–6.2	79.6	24–36	0.57–0.61
Stability phase	82–136	Synthetic vinasse	6.2–12.5	90.4	24–48	0.61–0.716

#### Start-up phase

The effective functioning of a UASB reactor depends on the development of a well-quality sludge blanket, which decline the period of the start-up step (Daud et al. 2018; Polizzi et al. 2022). During the start-up phase, the particulate sludges are mixed with the synthetic wastewater to promote sludge blanket formation and microorganism bioactivity. Synthetic wastewater is a carbon and nitrogen source and consists of potash fertilizer, sucrose, and urea. The sludge blanket enhances sludge settling capabilities, thereby increasing the reactor's resilience to loading rate shocks and inflow fluctuations and decreasing sludge washout (Acharya et al. 2011; Chen et al. 2017; Musa et al. 2018). Following the formation of the sludge blanket, synthetic vinasse progressively supplanted synthetic wastewater during the final seven days of strat-up phase (the 22nd to the 31st). This is due to the high susceptibility of anaerobic reactors to toxic compounds, which can disrupt the functionality of methanogen bacteria and degrade microorganisms (Musa et al. 2018). If synthetic vinasse is gradually fed into the UASB reactor, the sludge blanket is able to acclimate to the hazardous conditions progressively. Subsequently, when synthetic vinasse treatment commences, the sludge blanket is able to manage the elevated OLR.

#### Adaptation phase

**Fig. 2:**
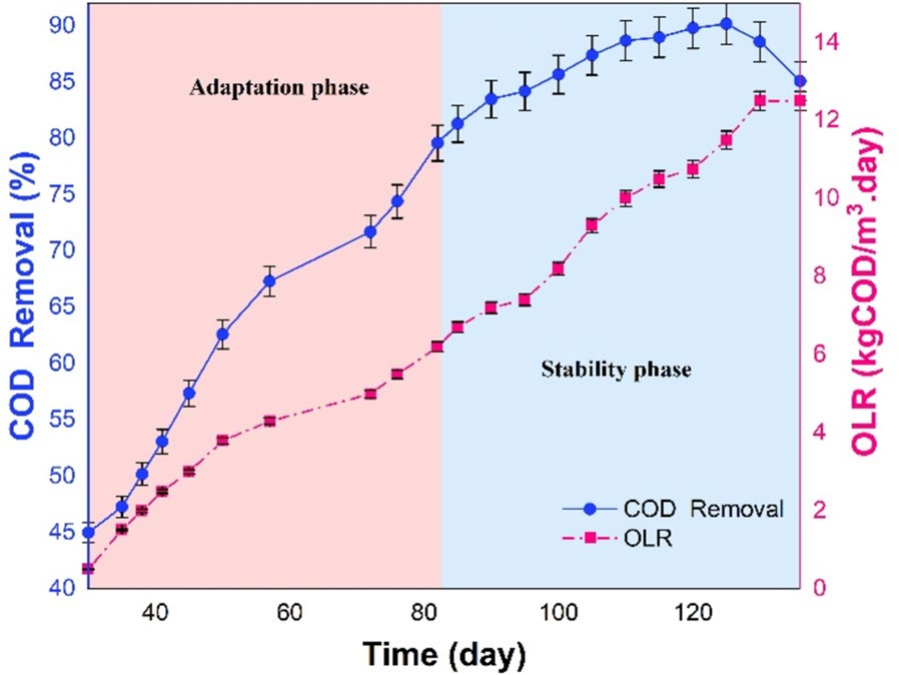
COD removal efficiency of the UASB reactor

The OLR for the UASB reactor at the beginning of the adaptation phase was 1.5 kgCOD/m^3^ day, where COD removal efficiency reached 47.3% at 31 °C (Fig. 2, days 30 to 82). The OLR was then gradually raised to 5.1 kgCOD/m^3^ day. The UASB reactor was consistent in improving COD removal till the 72th day, achieving an efficiency of 71.8%. Lastly, the OLR enhanced to 6.2 kgCOD/m^3^ day on the 82nd day and the COD removal efficiency steadily improved to about 80%.

Figure 2 shows the COD removal efficiency increases as the OLR increases during both adaptation and stability phases. The COD removal efficiency is expected to decrease with a sudden increase in OLR. However, the COD removal efficiency increased because the microorganisms adjusted to the toxic environment. HRT is a crucial factor in determining the stability of the reactor, the metabolic activity of microorganisms, and the reproduction of methanogenic bacteria and archaea (Xu et al. 2018). Reducing HRT results in biomass washout and reduces efficacy of suspended solids removal (Bhattacharya et al. 2017; Mainardis et al. 2020). In this phase, the optimal HRT was approximately 24 h. Once the reactor reached its stable state, it was capable of efficiently managing increased OLR.

#### Stability phase

Figure 2 depicts the outcome of the COD removal efficiency in the stability phase (Days 82 to 136). The reactor’s maximum loading capacity was ascertained by progressively augmenting the OLR from 6.2 to 12.5 kgCOD/m^3^.day. When the OLR was increased to 9.3 kgCOD/m^3^.day, the UASB achieved a COD removal efficiency of 87.4% on the 105th. On the 125th day, the OLR reached its peak value of 12.5 kgCOD/m^3^ day, representing an approximate 90.4% maximum COD removal efficiency. When the OLR surpassed 11.5 kgCOD/m^3^.day, a significant declination occurred in the COD removal efficiency.

Figure 2 illustrates a clear relation between the rise in OLR and COD removal efficiency. The findings indicate that the UASB reactor possesses the capability to withstand both high OLR and loading shock. The sludge blanket’s well-quality microbial community is responsible for the above mentioned UASB reactor capability, as it regulates biomass concentration and averts clogging issues. As the OLR was enhanced to 11.5 kgCOD/m^3^.day, the microorganisms consumed more food. Subsequently, the substrate degraded, and adaptation occurred gradually, leading to a rise in the COD removal efficiency (90.4%). This approach is supported by the studies of Alves et al., which developed a strategy of gradually adapting microorganisms to vinasse and achieved good results (Alves et al. 2023). However, when the OLR surpassed 11.5 kgCOD/m^3^.day, the COD removal deteriorated. A potential factor contributing to the reduction in COD removal efficiency is the overloading of the sludge blanket with noxious substances such as ammonia, sulfides, and heavy metals (Wainaina et al. 2019; Nogueira et al. 2021; Alves et al. 2023). Consequently, the microorganisms are rendered incapable of withstanding the excessive toxicity, which impairs their methanogenic activity and causes the sludge blanket to deteriorate, which then reduces COD removal efficiency to 85.1%. This study’s findings align with those of other researchers (Del Nery et al. 2018; Madaleno et al. 2020; Alves et al. 2023). Therefore, the optimal loading rate of the UASB reactor was determined to be 11.5 kgCOD/m^3^.day. An additional factor contributing to the reduction of the COD removal efficiency was the exacerbation of sludge floatation and excessive white foam formation within the reactor due to the rise in OLR. The yeast-like bacteria and anaerobic rod-shaped microorganisms proliferate on the wastewater's surface to generate foam. These operations lead to variations in tension and a thickening of the flow (Mariraj Mohan and Swathi 2022). In addition, the presence of gas bubbles which transport sludge fragments upward to the surface and subsequently cause enhanced sludge washout, may contribute to sludge flotation (Lettinga et al. 1980). Subsequently, an imbalance arises between the microbial community and anaerobic digestion within the reactor, causing biological processes to lose their stability. This instability of these biological processes ultimately culminates in a decline in the COD removal efficiency and the generation of methane (Utami et al. 2016).

Further variables that can impact the functioning of UASB reactors include the properties of the wastewater, pH levels, and HRT (Daud et al. 2018; Cecconet et al. 2022). An HRT of 3–72 h is selected for the UASB reactor to achieve a COD removal efficiency exceeding 80%. HRTs exceeding 24 h are used to purify high-strength effluent and extend contact time with biomass (Chong et al. 2012; Utami et al. 2016; Mariraj Mohan and Swathi 2022). Optimal HRT was achieved within a suitable time frame between 24 and 48 h in our research. This time range corresponds to the HRT range for vinasse treatment, as reported by other researches (Utami et al. 2016; Santiago-Díaz et al. 2019). Additionally, HRT directly correlates with Upflow Velocity (Vup). Optimal Vup ensures sufficient interaction between microorganisms and substrate, thereby reducing the formation of gas pockets, biomass loss, and the removal of total suspended solids (TSS) (Daud et al. 2018). The recommended range for Vup is typically between 0.5 and 1.5 m/h (Mariraj Mohan and Swathi 2022). In this study, Vup was maintained within the range of 0.430–0.716 m/h (Table 3). Moreover, the most suitable pH range for anaerobic digestion, particularly methanogenesis, is between 7.0 ± 0.1 and 7.2 ± 0.2. This range yields a COD removal efficiency exceeding 80% (Abdelgadir et al. 2014; Daud et al. 2018; Musa et al. 2018). The pH of the UASB reactor effluent remained within the range of 6.4 ± 0.1 to 7.6 ± 0.3. Throughout previous research, this increase in pH has been associated with improved COD removal efficiency (Taconi et al. 2008; Musa et al. 2018).

#### pH of the UASB reactor

**Fig. 3:**
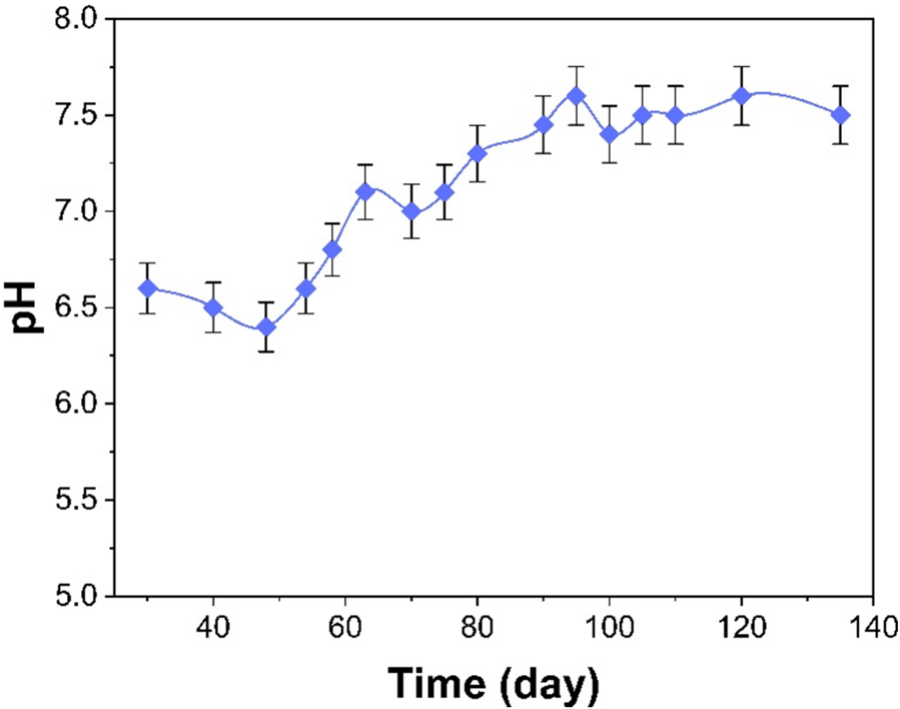
Evaluation of pH in UASB reactor

Figure 3 illustrates the pH change within the UASB reactor. The pH is a main parameter in anaerobic biological treatment as it affects the development of the microbial community (Nogueira et al. 2021). The low pH inflow disrupts the performance of methanogens by interfering with the cell walls of microorganisms (Jiraprasertwong et al. 2021). The ideal pH range for anaerobic treatment, particularly methanogenesis, is between 7.0 and 7.5 (de Santi Caraça et al. 2023; Tibúrcio Neto et al. 2024). This range achieves a COD removal efficiency that exceeds 80%. However, anaerobic digestion can operate efficiently within the pH range of approximately 6.6–7.6 (Musa et al. 2018; Nogueira et al. 2021). The pH of the UASB reactor outflow remained within the range of 6.4 ± 0.12 to 7.6 ± 0.20 throughout the entire reactor operational period and it is a suitable range that facilitates effective biological processes. The findings revealed that the pH declined to 6.4 ± 0.12 on 48th day. This decline is caused by the actions of acid-producing bacteria acting on biodegradable organic substances, leading to the formation of volatile fatty acids (VFA_s_) by anaerobic fermentation (Wainaina et al. 2019).). The pH nonetheless rose gradually to 7.6 ± 0.20 on the 95th day. Microorganisms gradually acclimate to novel environmental conditions, which accounts for above increase in pH. Prior studies have documented that elevated pH levels result in enhanced efficacy with regard to the removal of COD and the methane generation (Musa et al. 2018). The pH was eventually stabilized between 7.1 ± 0.14 and 7.6 ± 0.20 as a result of methane production.

Organic matter and pathogens are not entirely eradicated from high-strength wastewater by UASB reactors. Therefore, further post-treatment of the UASB reactor is generally needed to decrease the concentration of COD in the final outflow. With an average COD concentration of 1454.31 ± 5.17 mg/L in the UASB effluent, post-treatment is necessary to improve the quality of the outflow. In pursuit of this objective, a post-treatment strategy known as “modified Bardenpho process” was implemented, integrating the benefits of both aerobic and anaerobic processes.

### Assessment of the MBP performance

Despite the UASB reactor serving as the primary phase for COD removal, the UASB effluent contained a COD concentration that surpassed 1400 mg/L. The increase in outflow concentration of the UASB reactor can be ascribed to the concurrent enhancement in TN concentration and pH rate in the reactor. This adversely affects the COD removal efficiency. At precisely the same time that TN concentration of the UASB reactor outflow rose from 272 mg N/L to approximately 452 mg N/L, and the pH enhanced from an average of 7.0 ± 0.14 to 7.6 ± 0.20, the COD removal decreased. Therefore, TN and any remaining COD concentration must be eliminated concurrently. The UASB- effluent was treated using a five-stage Bardenpho process. This process used the residual COD as an electron donor (oxygen donor) to facilitate the decline of nitrite and nitrate nitrogen to N_2_ during the denitrification step and phosphorus removal during the aerobic stage. As a result, the overall efficiency of COD removal was enhanced.

#### COD removal efficiency

**Fig. 4:**
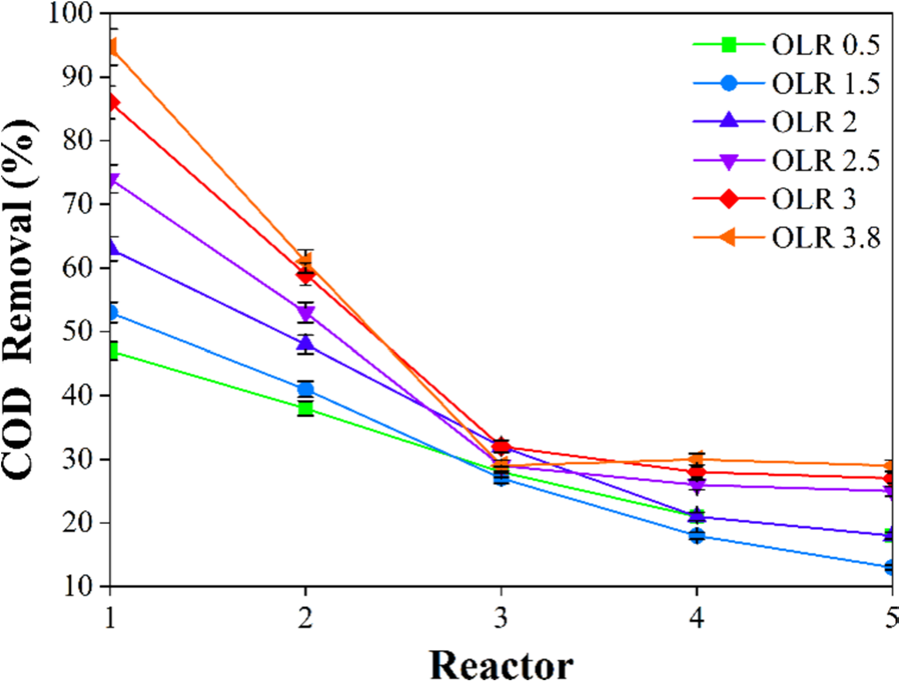
The COD removal efficiency of each reactor in the MBP at different OLRs

The COD removal efficiency was shown in Fig. 4.

The initial anaerobic stage of the process involves the main reactor, which is supplied with primary effluent and RAS. This stage of the process aims at converting hardly biodegradable organics into easily biodegradable organics for subsequent processes. This stage accounted for 53% of the total COD removal at a low OLR (1.5 kgCOD/m^3^.day), whereas the contributions from the second, third, fourth, and fifth reactors were 41%, 28%, 18%, and 13%, respectively (Fig. 4). As it is shown in Fig. 4, when OLR increases (2–3.8 kgCOD/m^3^.day), it will lead to an increased amount of nutrients reaching the microorganisms in all reactors. The COD removal efficiency subsequently escalated, culminating in a peak at 94.7%.

During the five-stage Bardenpho process, the anaerobic tank is strategically positioned to efficiently degrade carbon sources present in the inflow. However, this tank limits the denitrification efficiency, which is contingent upon the reflux ratio and typically ranges from 50 to 80% (Wang et al. 2023). When the anoxic stage is supplied with nitrate reflux from the aerobic step, denitrification will take place. Notwithstanding the elimination of COD during this process, nitrite and nitrate nitrogen (NO₂-N and NO₃-N) are transformed into nitrogen gas (N₂). Consequently, this reactor produced the greatest proportion of NH_4_^+^-N, which enhanced COD removal efficiency (refer to Fig. 7 and Sect. 3.3.1). Nitrification and the degradation of remaining COD were achieved in the second aerobic reactor by the activity of nitrate-reducing bacteria. Consequently, nitrite and nitrate concentrations are recycled to the anoxic reactor for the initial denitrification process. In addition, the phosphorus was removed by this reactor.

The second anoxic stage of the process performs further denitrification consuming the nitrate generated in the aerobic stage. The nitrate is an electron acceptor and the organic carbon, which is obtained from cell mass autophagy, is considered as an electron donor. The nitrogen and phosphorus gasses released from the solution during the final aerobic phase reduced the nitrogen gas level to below 8 mg/L and the phosphorus gas level to below 0.3 mg/L in the settlement tank. The release of nitrogen and phosphorus gasses is achieved by heterotrophic bacteria, such as phosphorus-accumulating organisms (PAOs), which are responsible for the accumulation of soluble phosphorus (PO^3^_4_-P) in biomass. In addition, the objective of the second aerobic reactor is to oxygenate the sludge to avoid its bulking. This is because the effluent of the second anoxic reactor contains almost no DO, which leads to deteriorated sludge settling properties. Consequently, a secondary aerobic reactor was used before the settling tank.

In conclusion, the effluent from the settling tank contained an estimated 41 mg/L of COD, which significantly meets the environmental discharge standard requirements. The HRT for each stage of the five-stage Bardenpho process falls typically the following ranges: 0.5–1 h for the anaerobic reactor, 1–2 h for the first anoxic reactor, 2–4 h for the second anoxic reactor, and 0.5–1 h for the second aerobic reactor. Normally, the system retains its SRT for a period of 10 to 25 days.

#### pH of the MBP

**Fig. 5:**
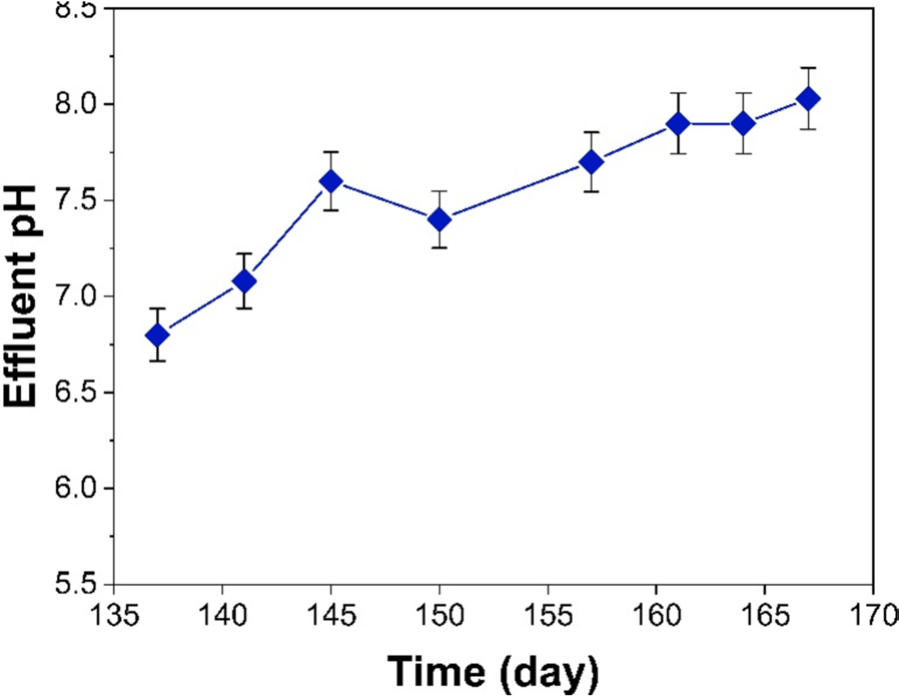
Assessment of variations in pH of the MBP

Figure 5 displays variations in the pH of the five-stage Bardenpho process (MBP).

Variations in reactor pH have an impact on the functioning of microorganisms and anaerobic digestion, subsequently influencing the COD removal efficiency and biogas generation. The acidogens bacteria exhibit predominance in the initial reactor and flourish in acidic environments with pH values ranging from 6.8 ± 0.13 to 7.1 ± 0.14. On the contrary, as the system operation progresses, the pH levels rise until they reach their maximum in the final stage. The mean pH of the effluent from stage 5 was approximately 7.6 ± 0.15, whereas the mean pH of the influent was approximately 6.8 ± 0.13. Following reflux dilution, the pH of the inflow to the system varied from 6.7 ± 0.13 to 7.04 ± 0.14, which was unquestionably a favorable conditions for microorganisms. A similar conclusion was obtained by Esfahani et al. (Banayan Esfahani et al. 2019). They find out that the denitrification process is most effectively at a pH level ranging from 7.0 to 8.0. Furthermore, the P uptake rate increased in a distinct linear fashion up to pH 8.0 (Nguyen et al. 2023).

### COD removal of the integrated UASB–MBP system

**Fig. 6:**
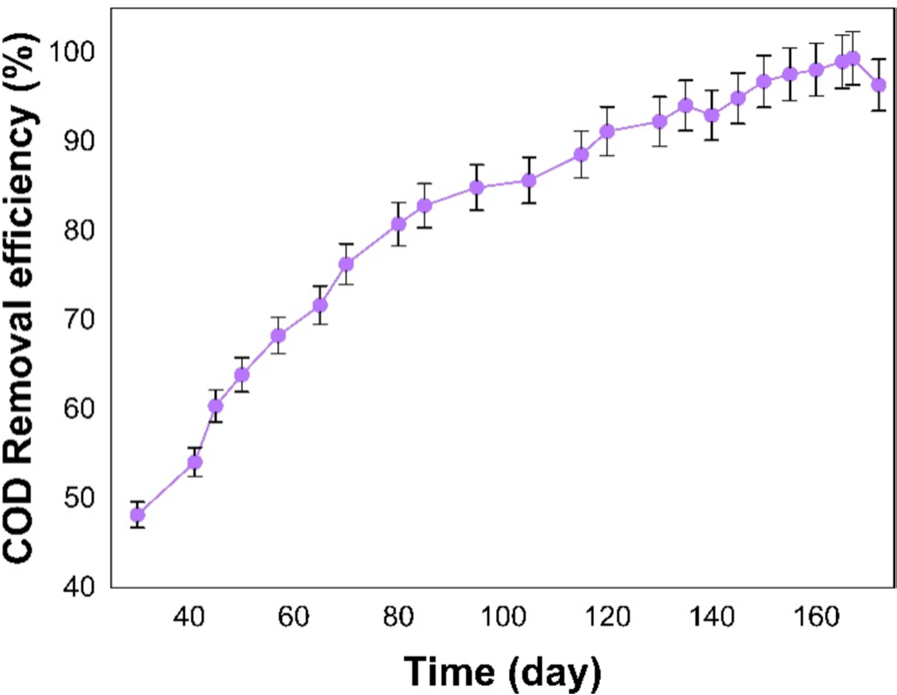
COD removal efficiency of the integrated UASB–MBP system

Figure 6 illustrates the overall COD efficiency of the UASB-MBP system.

Figure 6 shows the COD removal efficiency increased progressively. The UASB reactor is capable of achieving a peak COD removal efficiency of 90.2%. The subsequent MBP process improved the COD removal efficiency, achieving a total COD removal efficiency of 99.4% on the 167th day. Table 4 abridge the efficiency of the UASB-MBP system.

**Table 4:** Summary of the integrated system performance

Stages	UASB Reactor	modified Bardenpho process (MBP)	Integrated system
influent (mg/L)	14,952.43 ± 11.14	1454.31 ± 5.17	14,952.43 ± 11.14
effluent (mg/L)	1454.31 ± 5.17	76.41 ± 3.61	76.41 ± 3.61
COD removal	90.2%	94.7%	99.4%

The UASB bioreactor served as the primary phase for COD removal in the process of synthetic vinasse treatment. This is because the effective formation of the sludge blanket and the progressive acclimatization of bacteria to synthetic vinasse, which enables the UASB reactor to process raised OLR and achieves an approximate COD removal efficiency of 90.2%, occur in the primary phase. However, the COD concentration of UASB effluent exceeded 1400 mg/L. The effectiveness of the integrated system was impacted by the residual COD as an electron donor (oxygen donor) to decrease conversion of nitrite nitrogen and nitrate nitrogen to N_2_ during the denitrification step of the Bardenpho process, thereby causing a simultaneous decline in TN and COD concentration. The significant reduction in vinasse COD concentration is illustrated in Table 4. The settling tank effluent COD concentration and the overall COD removal efficiency of the UASB-MBP are 99.4% and 76.41 ± 3.61 mg/L, respectively. These efficiencies results exceed those of other studies in terms of COD removal efficiency in vinasse treatment (Ashrafi et al. 2019; Reis et al. 2019). Low COD in UASB-MBP effluent eliminates the need for costly additional treatments.

Although ammonia is necessary for bacterial growth as an essential nutrient, elevated concentrations of ammonia can hinder methanogenesis in the anaerobic digestion process and reduce the COD removal efficiency (Yenigün and Demirel 2013; Bodur et al. 2024). Thus, ammonia is considered a potential inhibitor in anaerobic digestion, especially when engaged with complex types of substrates (Liu et al. 2015; Mlinar et al. 2022). If the treatment process is hindered by high concentrations of free ammonia nitrogen (FAN), recovery strategies including substrate dilution, dilution of reactor contents, pH adjustment, adjustment of substrate C/N ratio, and the exterior addition of substances such as activated carbon can be used to improve removal efficiency, enhance biogas, and ensure a stable and efficient process (Azami et al. 2012; Yenigün and Demirel 2013).

In this experiment, the observed outcome can be attributed to the recirculation of the outflow from the settling tank, which reduced COD concentrations and ammonium levels in the inflow to the UASB. This, in turn, mitigated the inhibitory effects of elevated NH₄⁺-N levels on anaerobic bacteria. Consequently, the methanogenic process was initiated in the UASB reactor, significantly enhancing its efficiency (Ji et al. 2020).

In addition, due to the Bardenpho system’s high capability, the TN concentration in the outflow was diminished. Therefore, the effluent reflux no longer resulted in a deterioration of UASB performance. As a consequence, the effluent contained a reduced concentration of COD, indicating an enhanced efficiency COD removal efficiency. This finding demonstrates that the integrated UASB-MBP system effectively meets environmental standards requirements, yielding particularly high-quality final effluent while simultaneously diminishing the expenditure associated with advanced wastewater treatment. The outcomes of this study are surpass those that were previously documented by Procópio et al. and Cabrera-Daz et al., who used a UASB reactor to treat vinasse and also utilizing both aerobic and anaerobic reactors for treating the effluent (Cabrera-Díaz et al. 2017; Procópio et al. 2022).

#### NH_4_^+^-N and TN removal efficiency

**Fig. 7:**
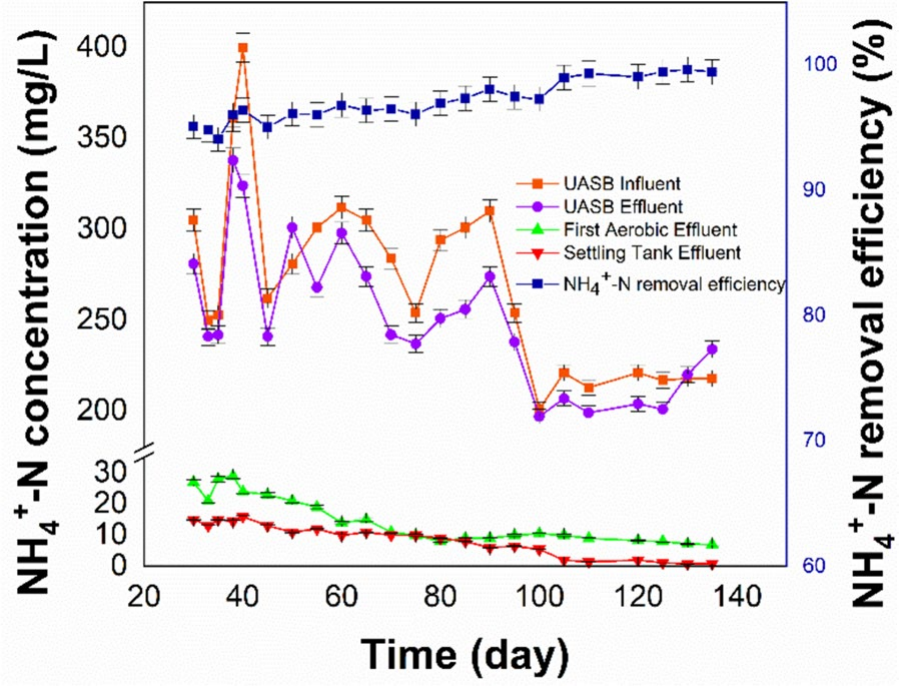
The NH_4_^+^-N concentration of the principal stages of the integrated UASB-MBP system removal efficiency

The key measure for controlling effluent in vinasse treatment is NH_4_^+^-N. This is due to the fact that an increase in concentration of NH_4_^+^-N inhibits anaerobic bacteria, which consequently diminishes the efficiency of COD removal (Liu et al. 2015). Various studies have proven that high levels of total ammonia nitrogen (TAN) inhibition, including free ammonia and ammonium nitrogen, may occur in the range of 1500–7000 mg/L, leading to failures in high-rate digesters (Yenigün and Demirel 2013; Liu et al. 2015; Wang et al. 2017a; Yang et al. 2019; Yan et al. 2020; Mlinar et al. 2022). McCarty and McKinney mentioned that the inhibition or toxicity primarily stems from free ammonia in solution rather than ammonium ions. They observed that anaerobic digestion completely ceases when the free ammonia nitrogen level reaches 150 mg/L. The cessation of anaerobic digestion affects gram-positive bacteria, which have less stable membranes and are susceptible to both NH₄⁺ and NH₃ (Yenigün and Demirel 2013; Bodur et al. 2024). Generally, it is recommended to maintain the NH_4_^+^-N concentration below 1000 mg/L to ensure effective wastewater treatment, especially in high-strength wastewater (Liu et al. 2015). Methanogenic archaea such as Methanosaeta and Methanosarcina, which contribute to biogas generation, are more vulnerable to ammonia than bacteria (Yan et al. 2020; Mlinar et al. 2022). Elevated TAN levels destabilize the process by increasing volatile fatty acid (VFA) accumulation, inhibiting microbial activity, and reducing methanogenesis rates, ultimately resulting in anaerobic digestion failure (Yang et al. 2019; Tsegaye and Leta 2022; Cisneros de la Cueva et al. 2024). Therefore, enhancing the efficiency of NH_4_^+^-N removal is vital. Figure 7 show the NH_4_^+^-N concentration across the principal steps and the overall removal efficiency of the UASB-MBP system.

Figure 7 show that there is no substantial difference between the NH_4_^+^-N concentrations in the input and output of the UASB reactor. On various occasions, the NH_4_^+^-N concentration increased slightly in the effluent. This phenomenon can be ascribed to the oxidation of organic nitrogen to NH_4_^+^-N by anaerobic bacteria. These findings align with those of similar studies (Petta et al. 2017; Mlinar et al. 2022; Bodur et al. 2024). The NH_4_^+^-N nitrogen exists as ammonia and ammonium ions in the modified Bardenpho system. This ammonia ion traverses the first two zones with minimal alteration. The sludge ages adequately only in the third aerobic zone for full nitrification to occur. The ammonia undergoes oxidation to produce nitrates and nitrites. Once the nitrates reaches the anoxic zone, where DO is absent, the bacteria convert the nitrates to nitrogen gas by using organic carbon compounds as hydrogen donors. The resultant nitrogen escapes into the atmosphere. The aerobic reactor outflow had the highest concentration of NO₃-N, indicating that the nitrification process primarily occurs in the aerobic reactor during the modified Bardenpho process. UASB-MBP eliminated approximately 99.63% of NH_4_^+^-N. The NH_4_^+^-N concentration in the outflow from the settling tank, which meets the discharge standard requirements, varied between 0.8 and 1.2 mg/L. The NH_4_^+^-N and TN concentration should not exceed 1.5 mg/L and 10 mg/L, respectively as concentrations above the mentioned values do not meet the discharge standard requirements (Ong 2018; Bodur et al. 2024).

**Fig. 8:**
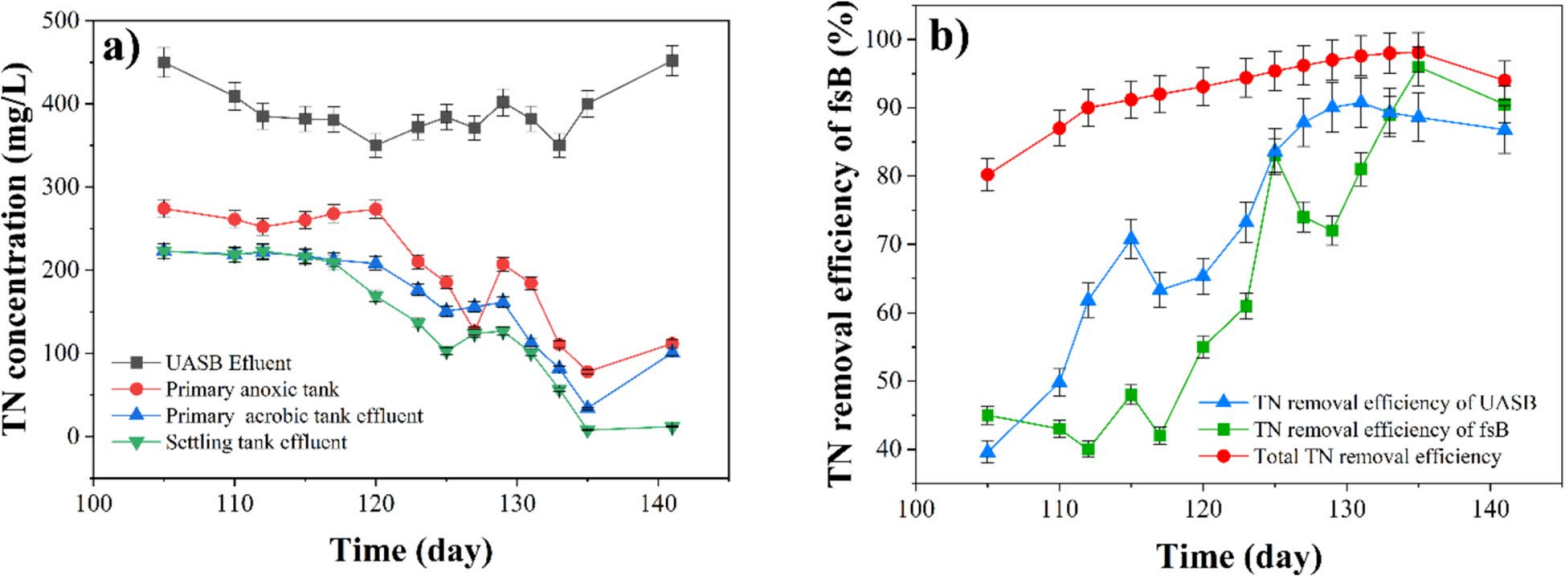
**a** The TN concentration of the main stages of the integrated UASB-MBP system, **b** the TN removal efficiency

Because the nitrogen in the vinasse is primarily exists as NH_4_^+^-N, with a notably high concentration (Lei et al. 2018), NH_4_^+^-N underwent oxidation to form nitrate-nitrogen in the aerobic tanks. Consequently, the high TN concentration could have been primarily caused by the nitrate-nitrogen concentration. Denitrification is the process by which nitrate-nitrogen is extracted from the anoxic tank (Bodur et al. 2024). Adequate amounts of organic carbon sources are required to donate enough electrons to transform nitrate-nitrogen to N_2_ during the denitrification process. As a result, the settling tank outflow had an estimated TN concentration of 8 mg/L (refer to Fig. 8a), which meets the discharge standard of less than 10 mg/L. The low TN concentration in the settling tank outflow can be ascribed to four factors (1) an appropriate HRT in the anoxic tank, (2) a nitrification reflux ratio of 200% to the first anoxic reactor, which guarantees complete conversion of TN to N_2_ and enhances the total denitrification efficiency of the system, (3) The DO concentration, which was retained at 3–5 mg/L in the aerobic tanks, was a vital factor in the nitrification process because it not only triggered the aerobic oxidation of COD but also facilitated the aerobic oxidation of NH_4_^+^-N (Ashrafi et al. 2019), and (4) Experimentation should be conducted at a mesophilic temperature, as temperatures exceeding 20 °C are optimal for phosphorus removal, nitrification, and denitrification (Maciołek et al. 2021; Bodur et al. 2024). Any decrease in wastewater temperature hinders denitrification. A decrease in temperature from 21 °C to 11 °C can cause a 75% decrease in the denitrification rate (Banayan Esfahani et al. 2019). These results are compatible with earlier investigations (Ashrafi et al. 2019; Bodur et al. 2024).

Carbon and nitrogen served as crucial energy sources for the proliferation of microorganisms throughout the process of wastewater treatment. Thus, the C/N ratio was frequently used to estimate the degree of organic matter decomposition (Wang et al. 2017b; Li et al. 2024). According to the findings of Fang et al. a C/N ratio below 2 is inadequate to support denitrification, leading to inadequate TN removal in the effluent (Fang et al. 2018). A high C/N ratio is thus advantageous for the elimination of nitrogen via denitrification (Li et al. 2024). In this research, the average C/N ratio was approximately 23, and the TN removal rate surpassed 99% (refer to Fig. 8b).

#### Phosphorous removal efficiency

**Fig. 9:**
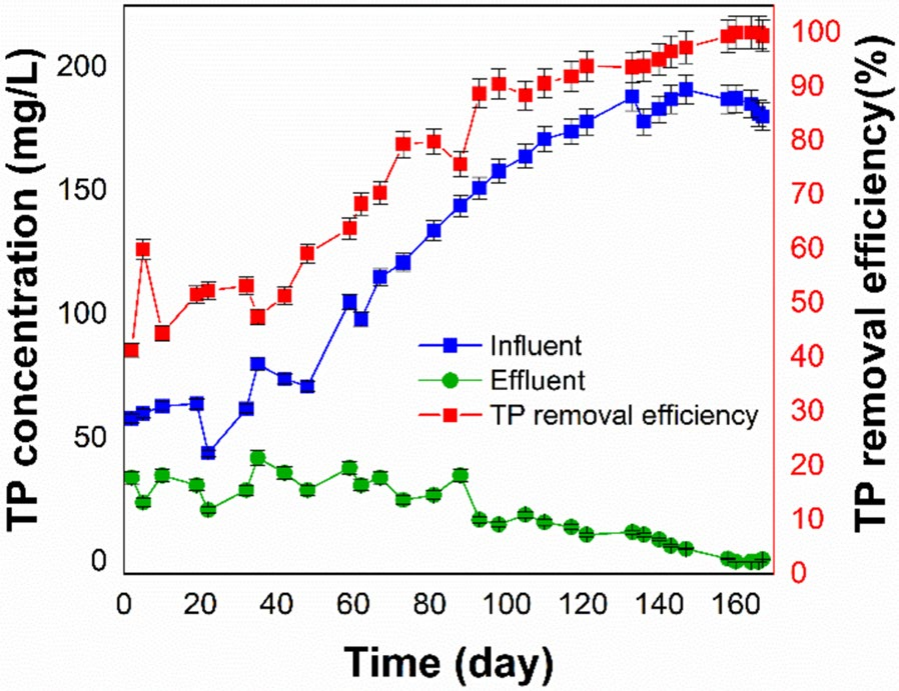
Total phosphorous removal efficiency of the integrated UASB-MBP system

Figure 9 show the phosphorus removal efficiency which was achieved by the integrated UASB-MBP system. The TP concentration of the influent wastewater decreased steadily, reaching 12.7 mg/L by the 10th day. The TP was gradually reduced in the ensuing days, and the outflow TP concentration was 0.012 mg/L when the TP removal efficiency reached its peak of 99.91%.

The MBP process achieves the highest phosphorus removal efficiency. Under anaerobic conditions, phosphorus is released by microbes in the MBP bioreactor. When the wastewater undergoes aerobic treatment, subsequently, the accumulating microbes absorb the phosphorus. During this stage, nitrifying bacteria oxidize the ammonia nitrogen. Then, the purified wastewater is introduced into the anoxic tank, where bacteria convert oxidized nitrogen into nitrogen gas to prevent the proliferation of phosphorus-limited bacteria. The presence of nitrate in the anoxic zone affects the release of phosphorus (Nengzi et al. 2018). The activation of heterotrophic bacteria, including phosphorus accumulating organisms (PAOs), occurs in the presence of nitrate in the anoxic zone. These bacteria accumulate soluble phosphorus (PO_4_^3^-P) in biomass, which is subsequently eliminated in the secondary aerobic stage. In addition, a significant portion of biodegradable organics is oxidized within this reactor. The phosphorus, which is assimilated by PAOs, eventually enters the sludge treatment system via the release of waste sludge. Total phosphorus (TP) levels in the anaerobic zone were slightly higher than those in the anoxic zone due to the presence of nitrate in the anoxic zone. This nitrate causes a constant release of phosphorus in the anaerobic zone. However, the lowest phosphorus content was found in second aerobic effluent (0.012 mg/L), where the TP removal efficiency was the highest (99.91%). One reason for the effluent lowest phosphorus content is that the effective denitrification efficiency minimizes the interference of recirculated nitrate in the release of anaerobic phosphorus. The external recirculation rate reaches around 200%. Another reason is the reduction of the active sludge retention time in the secondary settling tanks impacts the efficiency of phosphorus removal. Furthermore, the extension of HRT facilitated anaerobic phosphorus release, leading to an overall increase in phosphorus removal. The denitrification time can be increased by extending the HRT (Table 5), which leads to greater removal of nitrate and phosphorus. Table 5 shows the highest percentage of phosphorus removal in the two stages are: first anoxic stage at HRT 2 h (98.81%), first aerobic at HRT 8 h (90.89%), second anoxic stage at HRT 3 h (98.07%), and second aerobic stage at HRT 0.75 h (99.91%). Therefore, the highest phosphorus removal percentage occurs at the second aerobic stage at the HRT in range of 0.75 to 1 h.

**Table 5:** Total phosphorus removal efficiency at different HRTs

Stages	First anoxic		First aerobic			Second anoxic			Second aerobic		
HRT(h)	1	2	4	8	12	2	3	4	0.5	0.75	1
Total phosphorus removal (%)	95.14 ± 3.52	98.81 ± 2.01	95.54 ± 3.65	90.89 ± 4.36	96.31 ± 3.51	96.31 ± 3.41	98.07 ± 2.03	93.88 ± 4.23	94.76 ± 2.61	99.91 ± 2.93	95.99 ± 3.43

Based on literature data, when the COD/P ratio surpasses 50, process efficiency is suitable and the phosphorus concentration in the treated wastewater must not surpass 2 mg/dm^3^ (Maciołek et al. 2021; Li et al. 2024). Our results corroborate the findings by Maciołek et al. 2021 and Li et al. 2024. Because our treated wastewater phosphorus concentration remained below 0.02 mg/L. During operation, the average influent and effluent C: N: P ratios were 100:13:1.3 and 100:70:6.5, respectively. The C: N: P ratio for the anaerobic process should be 300:5:1, while for the aerobic process, it should be in the range of 100:5:1 to 100:10:1 (Del Nery et al. 2018; de Santi Caraça et al. 2023). However, at a COD/P ratio of 100/0.5, phosphorus began to appear in the effluent, where the average phosphorus removal efficiency dropped to 97%.

## Conclusion

The present study utilized an integrated UASB-modified Bardenpho system as an economically viable technology to enhance the bio-treatment efficacy of vinasse, which is a form of toxic industrial wastewater. To prevent the negative impact of high ammonium concentrations on anaerobic microorganisms, the reactor contents or substrate can be diluted, the influent pH adjusted by adding reagents, or the C: N ratio of the substrate modified. Therefore, recirculating the settling tank outflow reduced the concentration of NH_4_^+^-N and alleviated the inhibitory impact of great NH_4_^+^-N levels on anaerobic bacteria, thereby enhancing removal efficiency. Optimal operational conditions were achieved with an OLR of 11.5 kgCOD/m^3^ day, resulting in a COD removal efficiency of 99.41%. When the OLR exceeds 11.5 kgCOD/m^3^.day, the sludge blanket becomes overloaded with toxic compounds. As a result, the microorganisms are unable to endure high toxicity, causing the sludge blanket to deteriorate and reducing COD removal efficiency. Additionally, the modified Bardenpho process (MBP) plays a key role in improving TN removal efficiency, which can be enhanced by increasing the nitrification reflux ratio and extending the HRT of the primary anoxic tank, achieving an efficiency of 98.14%. The final output of the discharge was 51.06 mg/L, with NH_4_^+^-N and TN concentrations in the settling tank outflow ranging from 0.8–1.2 mg/L and 5.1–7.9 mg/L, respectively. Furthermore, the final TP removal efficiency was approximately 99.91%. The optimal performance occurred at HRT of 1.5, 2, 8, 3, and 1 h, respectively, in the anaerobic, first anoxic, first aerobic, second anoxic, and second aerobic compartments. The low COD concentration in the effluent from the Integrated UASB-MBP system obviates the need for additional costly advanced treatment of wastewater. An integrated UASB-MBP system significantly improves the COD removal efficiency and nutrient elimination during biological processes, and meets environmental discharge standard requirements and offers broad prospects for implementation in industrial wastewaters treatment.

The followings are recommended for further research:

- Assessment of the integrated UASB-MBP system’s performance in treating real vinasse.
- Assessment of the economic feasibility of this method compared with traditional methods.
- Evaluating the effect of NH_4_^+^-N on anaerobic digestion of vinasse and microbial community.

## Data Availability

All data generated or analyzed during this study are included in this article. The authors are willing to provide any additional data and materials related to this research that may be requested for research purposes.

## Abbreviations

COD: Chemical oxygen demand
BOD: Biological oxygen demand
TN: Total nitrogen
NH_4_^+^–N: Ammonia–nitrogen
TP: Total phosphorous
TSS: Total suspended solids
DO: Dissolved oxygen
UASB: Upflow anaerobic sludge blanket
MBP: Modified Bardenpho processe
HRT: Hydraulic retention time
VSS: Volatile suspended solids
MLSS: Mixed liquor suspended solids
MLVSS: Mixed liquor volatile suspended solids
